# Draft genomes of 10 *Vibrio cholerae* isolates collected in Sudan in 2019

**DOI:** 10.1128/mra.00376-24

**Published:** 2024-10-09

**Authors:** Muatsim Ahmed Mohammed Adam, Sam Sims, Amina Ahmed Awad, Luke Meredith, Basmat Abd Elhafeez Mohamed Ahmed, Rayan Adel Abdelrahman Ahmed, Hageer Alnazeer Ibrahim Saeed, Mohamed Abdalhafiz Alkhidir, Muna Idris Ahmed Abdelgadir, Hanan Ali Mohamed, Lubna Ahmed Badi, Yaiza Gutierrez Vazquez, Babak Afrough, Leena Inamdar, Angelika Kritz, Amal Barakat, Elamin Abualas, Shahinaz Ahmed Bedri

**Affiliations:** 1 Department of Genomics, National Public Health Laboratory (NPHL), Khartoum, Sudan; 2 New Variant Assessment Platform, UK Health Security Agency, London, United Kingdom; 3 Department of Bacteriology, National Public Health Laboratory (NPHL), Khartoum, Sudan; 4 Eastern Mediterranean Regional Office, World Health Organization, Cairo, Egypt; 5 National Influenza Center (NIC), National Public Health Laboratory (NPHL), Khartoum, Sudan; 6 Department of Molecular Biology, National Public Health Laboratory (NPHL), Khartoum, Sudan; 7 Sudan National Public Health Laboratory (NPHL), Khartoum, Sudan; Department of Biology, Queens College, Queens, New York, USA

**Keywords:** *Vibrio cholerae*, multidrug resistance (MDR), bacterial pathogen genomics

## Abstract

This report presents the first genomes from positive cases of cholera in Sudan. Genomic analysis of 10 *Vibrio cholerae* isolates, profiled as serogroup O1, reveals evidence of antimicrobial resistance genes and a 139-kb IncC plasmid with 99.74% identity to the multidrug-resistant plasmid pCNRVC190243 previously reported in Yemen and Lebanon.

## ANNOUNCEMENT

Sudan has experienced recurrent, perennial cholera outbreaks over the past five decades, including a recently declared outbreak (1). This report presents 10 draft genomes of *Vibrio cholerae* isolates obtained from patients presenting with acute watery diarrhea from across Sudan collected between 2 September and 3 December 2019 and submitted to the Sudan National Public Health Laboratory for sequencing.

Samples were cultured on Thiosulfate-Citrate-Bile Salts-Sucrose (TCBS) selective agar, and multiple colonies were stratified by sample and subjected to DNA isolation using the Zymobiomics Fungal/Bacterial DNA miniprep kit. Total nucleic acid from each sample was purified and concentrated using Zymobiomics Clean and Concentrator 5. The samples were barcoded and pooled at 300 ng each for nanopore sequencing library preparations using the Rapid Barcoding 96 kit (SQK-RBK110.96) following the manufacturer’s instructions. Bead purification was performed at a sample ratio of 1:1 with no targeted size selection of DNA. Pooled libraries were sequenced on a R.9.4.1 flow cell using the portable MinION MK1C (22.12.5). Base calling, demultiplexing, read quality filtering, and adaptor trimming were performed with Guppy v 6.4.2 on a fast mode with default settings.

Raw reads were assembled *de novo* [Flye v2.9.2 (2), using the –nano-raw flag], and draft contigs were polished [Medaka v1.8.0, Homopolish v0.4.1 (3, 4)]. Genome completeness was assessed with BUSCO v5.5.0 using the vibrionales_odb10 data set (5) and QUAST v5.2.0 (6). Assemblies were annotated by the NCBI Prokaryotic Genome Annotation Pipeline. Antimicrobial resistance (AMR) genes were predicted using abricate v0.8.13, against the NCBI database (7, 8). Genes appearing more than once were validated with BLAST v2.15 (9). Samples with a BUSCO completeness score >99% were selected for phylogenetic analysis. For phylogenetic inference, a representative set of 89 isolates was downloaded in raw read format from the ENA. Reads were aligned to the reference genome of *V. cholerae* O1 El Tor N16961, comprising Chromosome I (GenBank accession LT907989.1) and Chromosome II (LT907990.1), using minimap2 v2.26 (10). A whole-genome alignment with recombinogenic regions masked was constructed as previously described (11) with the following modifications: quality > 25, depth > 10, and mapping quality > 30. A maximum likelihood phylogenetic tree was constructed from 960 chromosomal single nucleotide variants (SNVs) with IQtree v2.2.6 (12) under the GTR model with 1,000 repetitions for bootstrapping. Default parameters were used for all software unless otherwise specified.

**Fig 1:**
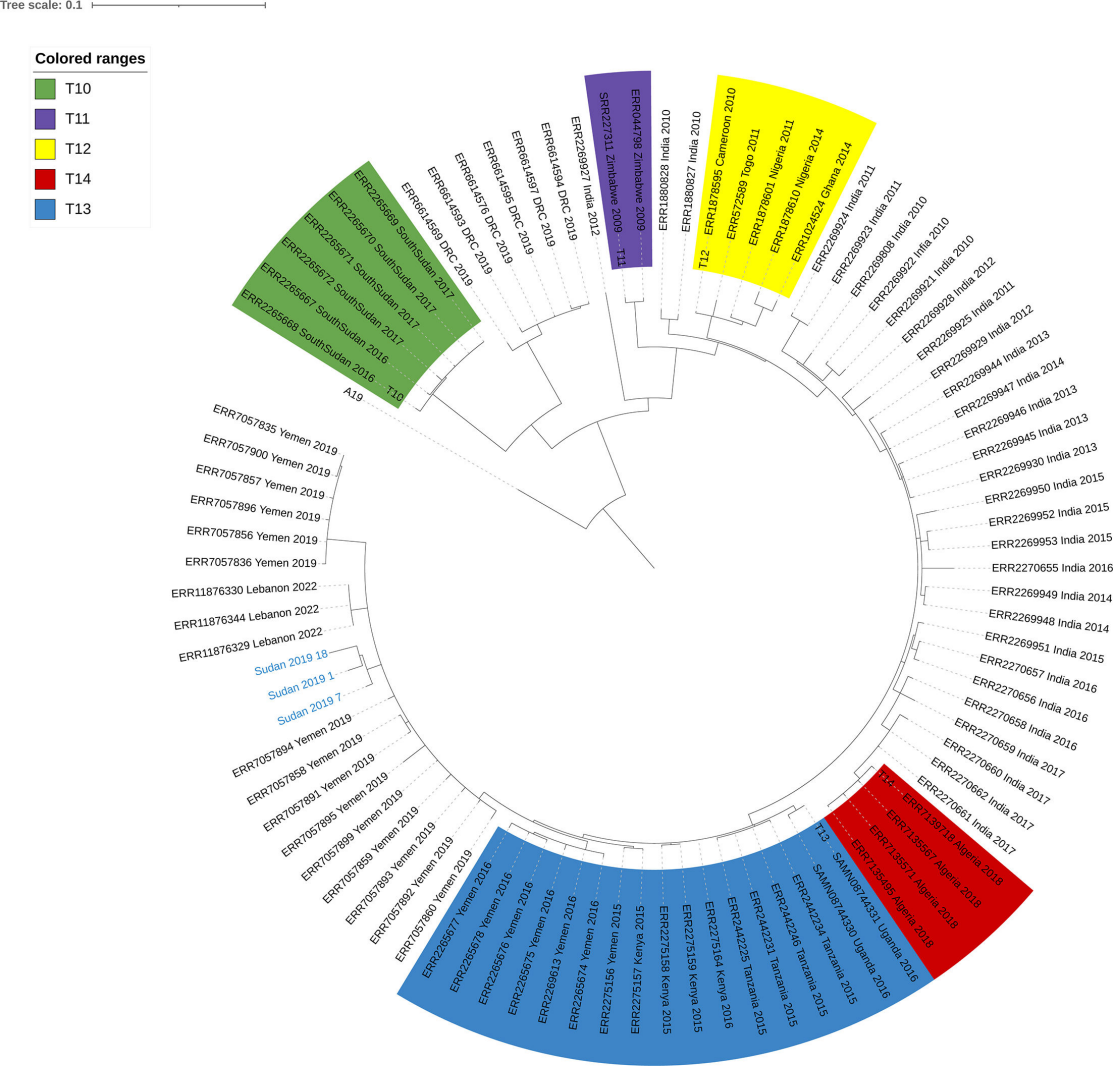
A maximum likelihood phylogenetic tree of the 92 genomic sequences included in this study, including A19, which was used as an outgroup. Clades are highlighted according to respective transmission events. Dark blue tip labels indicate Sudan isolates. For each contextual genome, the accession number, country of collection, and year of collection are shown on the tip labels.

The genome information for the 10 isolates is summarized in Table 1. All 10 isolates were identified as serogroup O1. Sudan_2019_1, Sudan_2019_5, and Sudan_2019_7 are predicted to carry dfrA1, varG, catB9, two copies of sul1, aadA2, blaPER-7, mph(E), msr(E), and mph(A), which encode for resistance to the following classes of antibiotics: trimethoprim, carbapenem, chloramphenicol, sulfonamide, streptomycin, cephalosporin, and macrolides. These three isolates carried an IncC plasmid of approximately 139 kb, which had a 99.74% nucleotide identity to the multidrug-resistant (MDR) plasmid pCNRVC190243 (GenBank OW443149.1) found in Yemen isolates collected between 2018 and 2019 (13) and more recently Lebanon (14). Although other isolates examined in this study exhibit a comparable AMR profile, they remain inconclusive due to fragmented or incomplete assemblies. Notably, Sudan_2019_18 was devoid of this plasmid. Additionally, phylogenomic analysis indicates a close relationship between the isolates studied and those collected in Yemen in 2019 (13) (Fig. 1). These findings establish a baseline understanding of *V. cholerae* diversity in Sudan and are helpful for monitoring the spread and control of MDR *V. cholerae* during the ongoing outbreak.

**TABLE 1:** Summary of samples used in this study

Sample ID	Region, province, district	Collection date (day/mo/yr)	No. of reads	Read *N*_50 (bp)_	Assembly *N*_50 (bp)_	Coverage (×)	Length (bp)	GC(%)	No. contigs	BUSCO completeness score (%)	Predicted antimicrobial resistance genes	GenBank accession no.	NCBI SRA accession no.
Sudan_2019_1	Blue Nile, Al-Roseries, Al-Roseries	02/09/2019	102,589	7,797	3,025,932	48	4,214,269	47.6%	3	100.0	dfrA1, varG, catB9, sul1, sul1, aadA2, blaPER-7, mph(E), msr(E), mph(A)	GCA_038031065.1	SRR28345621
Sudan_2019_2	Blue Nile, Al-Roseries, Al-Roseries	02/10/2019	121,991	1,556	105,571	23	4,201,963	47.7%	112	71.3	dfrA1, varG, sul1, aadA2, mph(E), msr(E), mph(A)	GCA_038031075.1	SRR28345620
Sudan_2019_3	Sennar, Abuhugar, Wadel Nayal	13/09/2019	120,555	6,118	582,734	43	4,090,608	47.6%	18	84.5	dfrA1, varG, sul1, aadA2, mph(E), msr(E), mph(A)	GCA_038031005.1	SRR28345619
Sudan_2019_4	Sennar, Abuhugar, Wadel Nayal	13/09/2019	187,090	2,835	223,714	40	4,186,610	47.6%	37	76.8	dfrA1, varG, catB9, sul1, aadA2, blaPER-7 mph(E), msr(E), mph(A)	GCA_038031015.1	SRR28345618
Sudan_2019_5	Blue Nile, Al-Damazien, Al-Shaty	09/11/2019	286,582	1,077	252,997	44	4,242,926	47.6%	43	79.2	dfrA1, varG, catB9, sul1, sul1, aadA2, blaPER-7, mph(E), msr(E), mph(A)	GCA_038031025.1	SRR28345617
Sudan_2019_6	Blue Nile, Al-Roseries, Al-Roseries	15/09/2019	157,356	848	29,056	21	4,042,406	47.7%	229	67.0	dfrA1, catB9, sul1, aadA2, blaPER-7,mph(E), msr(E), mph(A)	GCA_038030935.1	SRR28345616
Sudan_2019_7	Blue Nile, Al-Roseries, Al-Roseries	05/09/2019	262,453	7,192	3,040,641	98	4,230,319	47.6%	3	100.0	dfrA1, varG, catB9, sul1, sul1, aadA2, blaPER-7, mph(E), msr(E), mph(A)	GCA_038030955.1	SRR28345615
Sudan_2019_18	Sennar, Sennar, Sennar	23/09/2019	247,162	6,516	3,022,820	100	4,073,050	47.4%	2	99.4	dfrA1, varG, catB9	GCA_040412195.1	SRR28345614
Sudan_2019_19	Sennar, Sennar, Sennar	23/09/2019	202,685	4,809	3,031,942	97	4,241,633	47.6%	5	98.6	dfrA1, varG, catB9, sul1, aadA2, blaPER-7, mph(E), msr(E), mph(A)	GCA_038030905.1	SRR28345613
Sudan_2019_20	El-Gazeera, El-Gazeera, El-Gazeera	03/12/2019	121,104	3,234	339,515	31	4,184,397	47.6%	36	77.1	dfrA1, varG, Sul1, aadA2, blaPER-7, mph(E), msr(E), mph(A)	GCA_038030925.1	SRR28345612

## Data Availability

The complete genome sequences were deposited in GenBank, and raw reads were deposited in the SRA depository under the accession numbers listed in Table 1 (PRJNA1087979).
